# Supplementary material for: Identification of Freezing-Responsive microRNAs and Their Targets in Chinese Jujube by Small RNA and Degradome Sequencing
Source: Int J Mol Sci. 2026 Sep 4;27(17):7912. doi: 10.3390/ijms27177912 (PMC13566890; doi:10.3390/ijms27177912)
Supplement: Supplementary file 1 [file ijms-27-07912-s001.zip › Supplementary Material S2/novel_miR_99.pdf]

Provisional ID	:	novel_miR_99
Score total	:	85.8
Score for star read(s)	:	3.9
Score for read counts	:	79.1
Score for mfe	:	1.1
Score for randfold	:	1.6
Score for cons. seed	:	
Total read count	:	167
Mature read count	:	166
Loop read count	:	0
Star read count	:	1

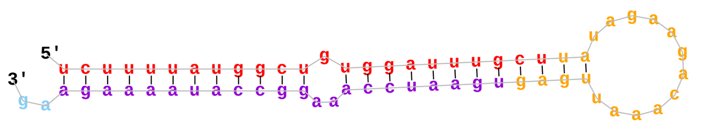

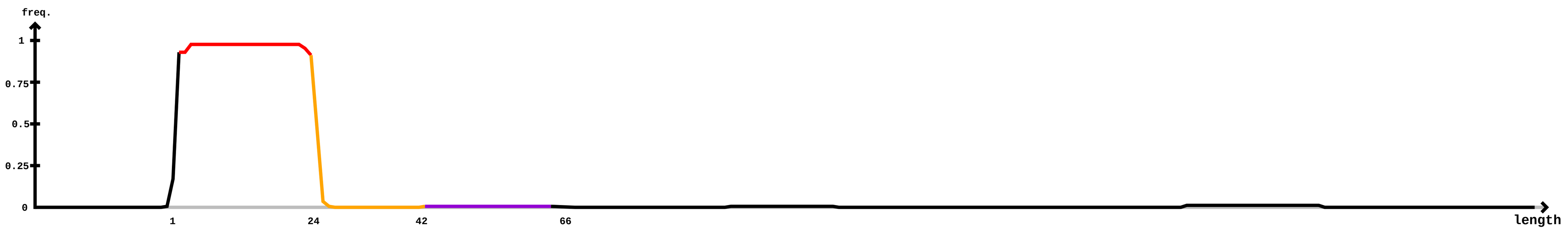

Mature Star

```
5'- ccuuacuuuaaaaguaaggacuucuuuuauggcuguggauuugcuuauagaagacaaauugagugaauccaaaggccauaaaagaaguuuuauuuagaaaauauaaagucaaccuaagaauagaccaacugcuauauaguuuugaucucuucagaaguaucucucaaauuauaaugggagauguaauaaguugaaaagugcaaagguuaucgcauucauguguagguuuguucguuuuaaguuaauaa -3' obs
    ccuuacuuuaaaaguaaggacuucuuuuauggcuguggauuugcuuauagaagacaaauugagugaauccaaaggccauaaaagaaguuuuauuuagaaaauauaaagucaaccuaagaauagaccaacugcuauauaguuuugaucucuucagaaguaucucucaaauuauaaugggagauguaauaaguugaaaagugcaaagguuaucgcauucauguguagguuuguucguuuuaaguuaauaa exp
    ((((((((...))))))))(((((((((((((.(((((((((((..........))))))))))))..))))))))))))))).........................(((((((((.(((((.(((...(((((..((.(((((...(((((((((.......)))))))))......))))).))..)))))...))).)))......)).)))))))))...............
```

read	reads	mm	sample
....................cuucuuuuauggcuguggauuugcuuau	1	0	JCK-3
.....................uucuuuuauggcuguggauuug	2	0	LT-3
.....................uucuuuuauggcuguggauuugc	2	0	LT-1
.....................uucuuuuauggcuguggauuugcu	1	0	JCK-1
.....................uucuuuuauggcuguggauuugcu	1	0	LT-1
.....................uucuuuuauggcuguggauuugcu	1	0	JCK-3
.....................uucuuuuauggcuguggauuugcu	8	0	LCK-3
.....................uucuuuuauggcuguggauuugcu	1	0	LT-3
.....................uucuuuuauggcuguggauuugcu	12	0	LT-2
......................ucuuuuauggcuguggauuug	2	0	LT-3
......................ucuuuuauggcuguggauuugc	1	0	JCK-3
......................ucuuuuauggcuguggauuugc	1	0	JCK-2
......................ucuuuuauggcuguggauuugc	2	0	LT-3
......................ucuuuuauggcuguggauuugc	1	0	JCK-1
......................ucuuuuauggcuguggauuugcu	20	0	JCK-1
......................ucuuuuauggcuguggauuugcu	25	0	LCK-3
......................ucuuuuauggcuguggauuugcu	12	0	LT-3
......................ucuuuuauggcuguggauuugcu	16	0	JCK-2
......................ucuuuuauggcuguggauuugcu	17	0	JCK-3
......................ucuuuuCuggcuguggauuugcu	1	1	JCK-2
......................ucuuuuauggcuguggauuugcu	11	0	LT-1
......................ucuuuuauggcuguggauuugcu	1	0	JT-3
......................ucuuuuauggcuguggauuugcu	1	0	JT-2
......................ucuuuuauggcuguggauuugcu	15	0	LT-2
......................ucuuuuauggcuguggauuugcuu	1	0	JCK-1
......................ucuuuuauggcuguggauuugcuu	1	0	JCK-2
......................ucuuuuauggcuguggauuugcuA	1	1	LT-1
........................uuuuauggcuguggauuugcu	1	0	JCK-2
........................uuuuauggcuguggauuugcu	1	0	JCK-3
........................uuuuauggcuguggauuugcuu	1	0	LT-3
........................uuuuauggcuguggauuugcuua	1	0	JCK-1
........................uuuuauggcuguggauuugcuua	1	0	JCK-2
........................uuuuauggcuguggauuugcuua	2	0	LT-1

```
                      Mature                                  Star
ccuuacuuuaaaaguaaggacuucuuuuauggcuguggauuugcuuauagaagacaaauugagugaauccaaaggccauaaaagaaguuuuuauuuagaaaauauaaagucaaccuaagaauagaccaacugcuauauaguuuugaucucuucagaaguaucucucaaauuauaaugggagauguaauaaguugaaaagugcaaaggguuaucgcauucauguguagguuuguucguuuuaaguuaauaa
........................uuuuauggcuguggauuugcuua...........................................................................................................................................................................................   1   0   LT-2
...............................................................ugaauccaaaggccauaaaaga.....................................................................................................................................................................   1   0   JCK-2
..................................................................................................................cuaagaauGgaccaacug......................................................................................................................   1   1   LT-3
..............................................................................................................................................................................................guugaaaagugcaaaggguuauc.....................................   2   0   LT-1
```
